# A Mie resonant antenna with high sensitivity for force and strain measurement

**DOI:** 10.1038/s41598-017-04911-2

**Published:** 2017-07-04

**Authors:** Lingling Wu, Xiaoqing Xi, Bo Li, Ji Zhou

**Affiliations:** 1 State Key Laboratory of New Ceramics and Fine Processing, School of Materials Science and Engineering, Tsinghua University, Beijing, 100084 China; 2 Advanced Materials Institute, Shenzhen Graduate School, Tsinghua University, Shenzhen, China

## Abstract

We demonstrate the experimental and simulated performances of a Mie resonant antenna for force sensing with high sensitivity for compressive force and strain measurements inside soft materials. The proposed sensor is compatible with biological specimens and has small dimensions. It comprises a pair of dielectric cubes and an elastic layer of silicone rubber. The applied force is determined by measuring the redshift of the operating frequency when a mechanical load applied. Both simulated and experimental results demonstrate that the relationship between the frequency shift of the sensor and the applied compressive load could be fitted well using a quadratic equation with a maximum fitting error less than 17%, which enables highly sensitive telemetric force measurements to be performed inside structures by observing the operating frequency shift. The proposed design provides a simple and low-cost approach to realizing highly-efficient telemetric measurement inside soft materials such as biological tissues.

## Introduction

Measuring force and strain via telemetric methods is highly desirable in many industries, including civil engineering, geology exploration and biomedical diagnosis^1, 2^. Electromagnetic (EM)-based sensors that measure applied forces by reading the operating frequency shift of EM devices, which are highly sensitive to mechanical loading, have been proposed to address this challenges^2–4^.

EM metamaterials are some of the first metamaterials studied and their remarkable properties have been the topic of numerous reports^5–9^. EM metamaterials have potential for application in a wide variety of areas, in particular for sensing^10–15^. Inspired by the metamaterial approach, researchers have designed various sensors to diagnose strain or force telemetrically^2–4, 16–19^. Previously reported strain- or force-measurement devices typically comprise metallic resonators, such as split-ring resonators (SRRs)^2–4^, or complex metallic nanostructures^18–22^. However, because SRR-based sensors with substrates such as silicon^2^ or Kapton tape^16^ have limited sensing and deformation ranges, they are only suitable for large force and small strain measurement, such as detecting the rigidity of buildings or bones. SRR-based sensors are not suitable for measurement of small forces and large strains, which commonly occur in soft materials such as biological tissues, because of the limited sensitivity and large size. Furthermore, the sensors usually need to be adhered to the surface of the tested materials, which can cause measurement errors and lead to difficulties in measuring objects with complicated structures. Recent studies have shown that either linear or nonlinear performance can be achieved by introducing elastomer materials such as polydimethylsiloxane into metal nanoparticle structures^19, 23–26^, which has enabled the development of flexible EM devices that can be mechanically tuned with large strain deformation and have broad applications in biomedical engineering. These nanostructures usually require complex fabrication processes and operate in high-frequency ranges, such as near-infrared wavelengths^19^ or in the visible region^21, 22^. Because lower EM frequencies are more easily transmitted in biological material than high frequencies, it is highly desirable to reduce the operating frequency of the metamaterial sensors. However, decreasing the frequency usually requires the dimensions of the metamaterial sensors to increase, which has a negative influence on the measured system and prevents their application in many industries, in particular in medical diagnosis. To measure force and strain inside complicated biological structures such as soft tissues and muscle, it is necessary to develop small, high-sensitivity, biologically-compatible sensors that are able to operate in the low-frequency region.

In this paper, we propose a dielectric Mie resonant sensor that operates in the microwave region and is able to detect small forces and large strains inside biological materials. The sensor has dimensions of 2 mm × 2 mm × 6 mm. Besides, it has deep transmission dips, which permits an accurate measurement of small forces, such as those that occur in joints, muscles and soft tissues.

## Results

**Figure 1:**
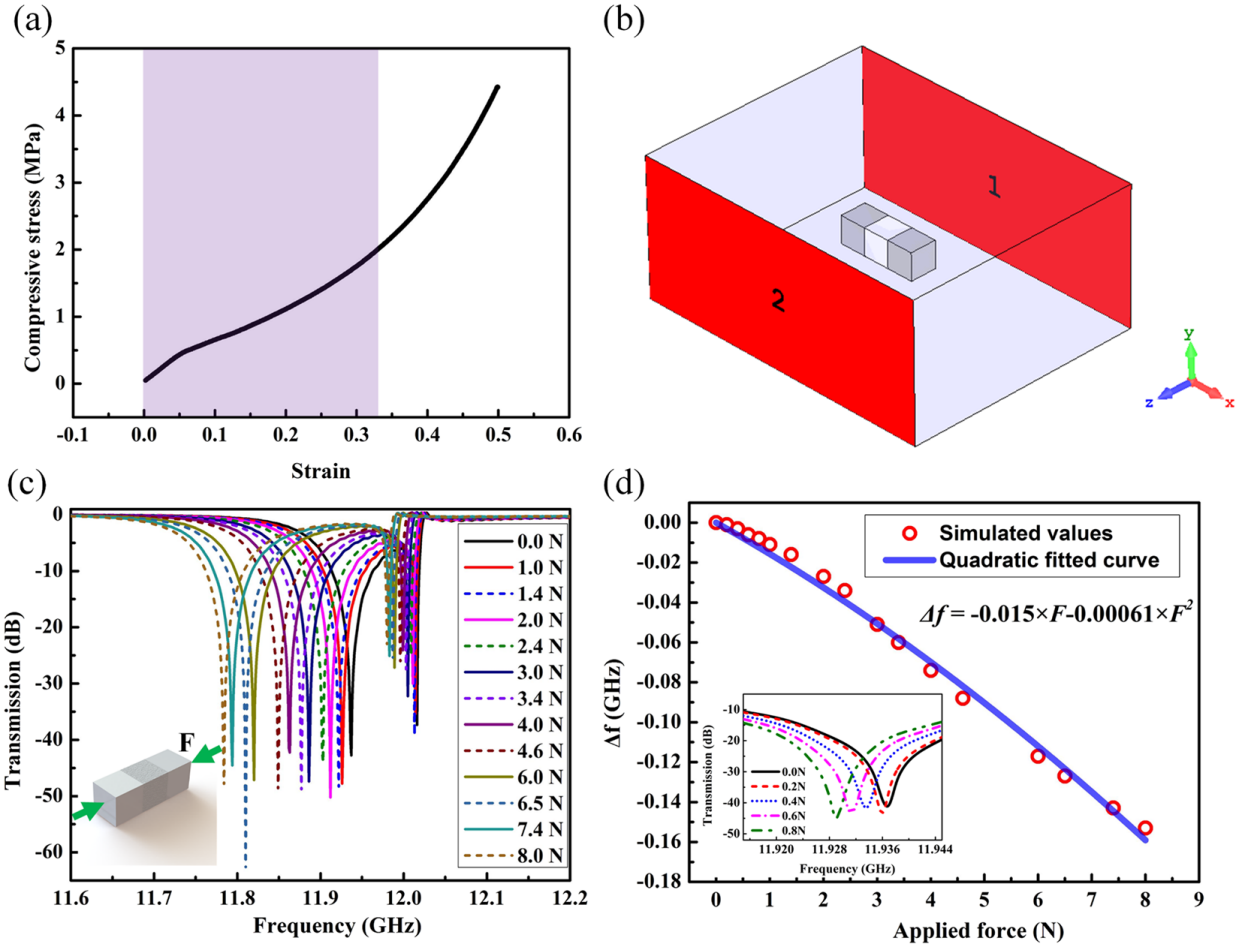
(**a**) The measured relationship between the compressive stress and strain for the silicone rubber. (**b**) The simulation setup in the CST microwave studio. (**c**) The simulated transmission spectra curves of the dielectric Mie resonant antenna under different compressive force levels. (**d**) The calculated relationship between the frequency shift and the applied force. The inset of (**d**) shows the simulated frequency shift at five loading levels from 0 N to 1 N.

The force-sensitive sensor was designed by combining a dielectric resonator and an elastomer layer. The dielectric resonator comprised two dielectric ceramic cubes, and the elastomer layer was fabricated from silicone rubber. The compressive stress–strain relationship of the silicone rubber was tested using a material testing machine (Zwick/Roell Z020, Germany) and the result is shown in Fig. 1(a). Our sensor is designed in a strain range from 0 to 0.33, as marked by the shading area in Fig. 1(a). From the figure, we can see that the rubber presents a slight non-linearity at the beginning of loading. To determine the reason for the nonlinear behavior, we performed 10 cycles of loading and unloading tests on the rubber. The results are shown in Fig. S1 in the supporting information, which present a permanent nonlinearity at the beginning of each loading process. We believe that it can be attributed to two sources: the first is the nonideal boundary conditions that apply during the measurement, which are unavoidable, and the second is the inherent nonlinearity of the rubber material. While we cannot determine which of the two is the main cause of the nonlinearity, we can conclude that the rubber material presented a permanent nonlinearity at the beginning of loading process. The hysteresis between the loading and unloading is derived from the viscoelasticity of the rubber, indicating that the rubber cannot recover to its original shape immediately after unloading. About this point, we will present in detail in the experimental section.

For a ceramic particle with fixed dimensions and permittivity, there will be a series of resonance dips in the transmission spectrum^9^. We used the transmission spectra in the S_21_ configuration to observe the frequency shift of the first Mie resonance dip because it is sharper than subsequent dips. The deformation of the rubber layer caused a redshift of the first Mie resonance frequency because of the coupling effect between dielectric cubes. Strong magnetic resonance occurs at the first Mie resonance frequency and the dielectric cubes can be considered as magnetic dipoles. When compressive force is applied to the dielectric cubes, the interplay between the magnetic dipoles becomes stronger and leads to an increase of the resonance frequency^27–29^.

To explore the relationship between the applied force and the first Mie resonance frequency shift of the sensor, we performed simulations using the Microwave Studio software package (CST Studio Suite 2015, Germany). The system was modelled as a rectangle having dimensions 22.86 mm × 10.16 mm and surrounded by a perfect conductor, as shown in Fig. 1(b). The permittivity of the dielectric cubes (CaTiO_3_–2 wt% ZrO_2_ ceramic) was set to 115 at room temperature and the permittivity of the intermediate layer was set to 2.3, to coincide with the experimental parameters. The dimensions of the dielectric cubes and the elastic layer were 2 mm × 2 mm × 2 mm. A compressive force between 0.0 N and 8.0 N was applied in the direction orthogonal to the dielectric cubes, as shown in the inset of Fig. 1(c). Because the Young’s modulus of ceramic cubes was much higher than that of the silicone rubber, their deformation under the compressive force was neglected in both the simulation and experiment.

The simulated frequency shift of the sensor with different compressive forces applied is shown in Fig. 1(c). The first resonance frequency of the dielectric sensor experienced a redshift as the applied load increased. From the simulated curves shown in Fig. 1(c), we obtained the relationship between the frequency shift (denoted as Δ*f*) and the applied force, as shown in Fig. 1(d). The magnitude of the frequency shift of the sensor increased with the applied force, which means that the applied force can be determined telemetrically by observing the redshift of the operating frequency. From Fig. 1(a) and Fig. S1, we can see that the rubber layer always presents a non-linearity mechanical performance at the beginning of loading. Therefore, we decided to use a nonlinear method to analyze the relationship between the frequency shift and the applied compressive force. We found that the simulated values could be fitted well using a quadratic equation, as shown in Fig. 1(d). From the simulated results, we can conclude that the silicone rubber is very sensitive to mechanical loads and experiences a large frequency shift under small loading levels.

**Figure 2:**
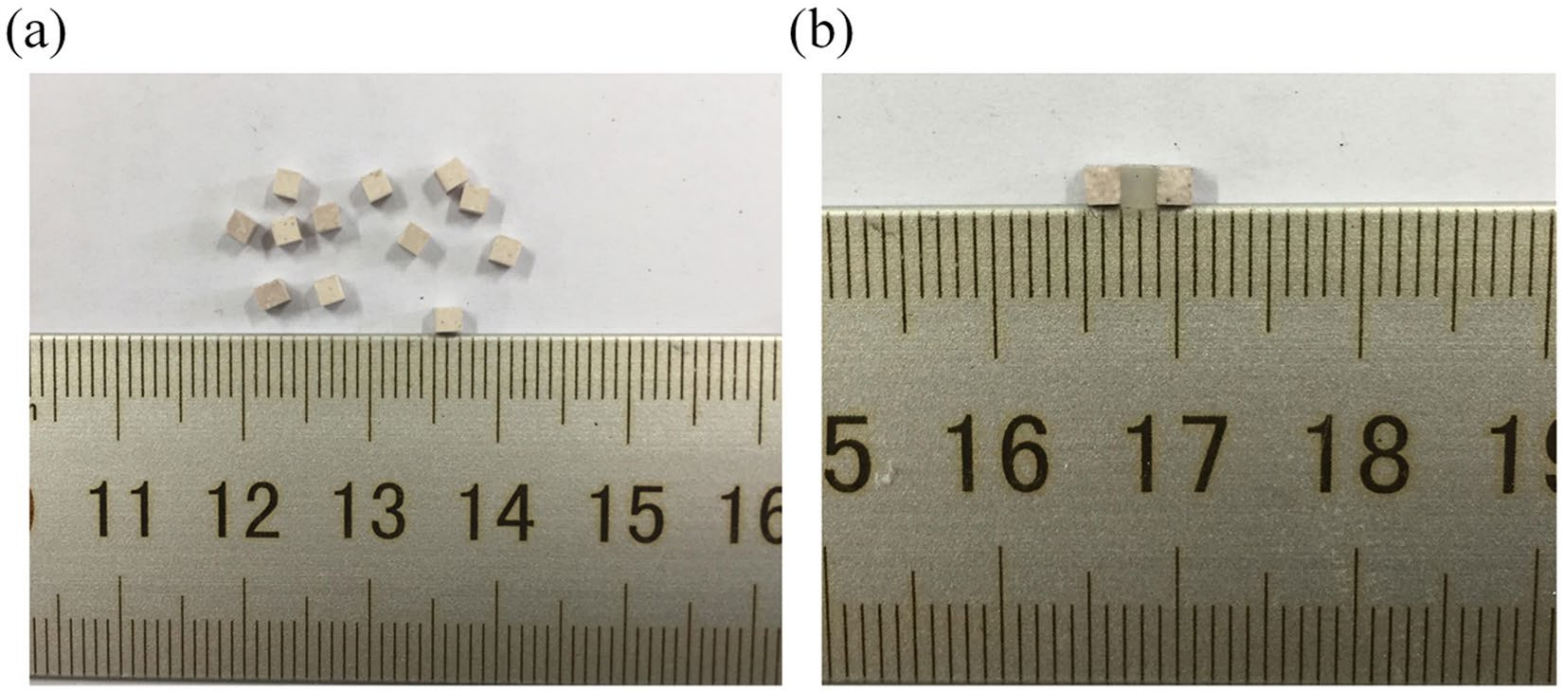
(**a**) The dielectric ceramic cubes with dimensions of 2 mm × 2 mm × 2 mm. (**b**) The fabricated force sensor composed of two dielectric cubes and a silicone rubber layer.

To demonstrate the performance of the designed force sensor experimentally, we chose ceramic cubes as the dielectric resonators and silicone rubber as the elastic layer, as in the simulation. Figure 2(a) shows the dielectric cubes with dimensions of 2 mm × 2 mm × 2 mm. The main constituent of the dielectric cubes was calcium titanate (CaTiO_3_), which has been shown to be biologically compatible and has been widely used as a biomedical material^30, 31^. The dielectric cubes had a permittivity of approximately 115 and a loss tangent of 0.001 at room temperature. This allows the cubes to be used to make high-permittivity and low-loss sub-wavelength resonators with a high quality factor, which are suitable for operation in the microwave bands. The dielectric sensor was fabricated by bonding two dielectric cubes with a silicone rubber intermediate layer with the same dimensions, as shown in Fig. 2(b). The total size of the sample was 2 mm × 2 mm × 6 mm.

**Figure 3:**
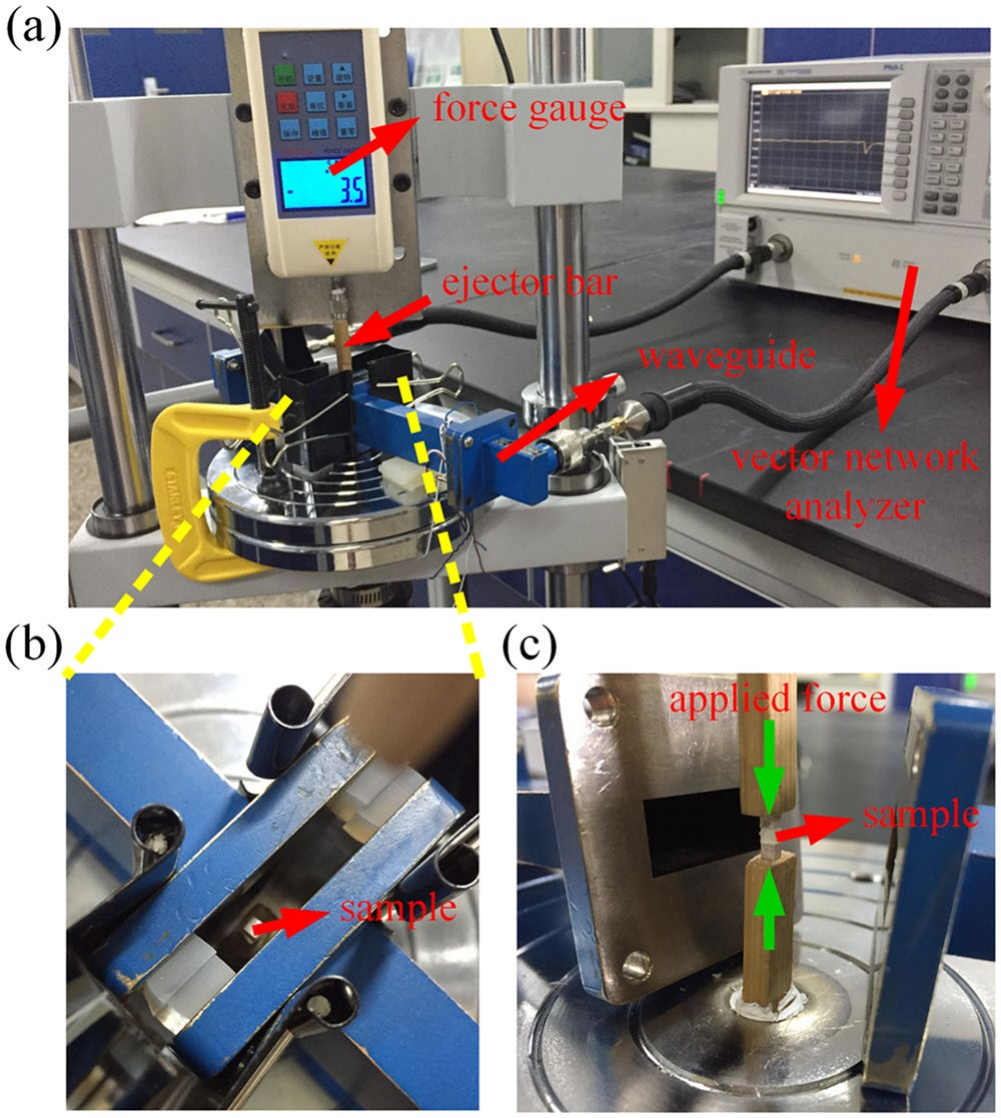
(**a**) An overview of the experiment setup. (**b**) The sample placed on the wood ejector bar. (**c**) The compressive force applied on the sensor orthogonally by the ejector bars.

To measure the transmission performance of the sensor under different mechanical loading levels, we constructed an experimental setup that combined a material testing machine with a vector network analyser (Agilent PNA-LN5230C), as shown in Fig. 3(a). A force gauge was used to record the compressive force applied on the sensor. To minimize the measurement error, we used two wood sticks, which had a dielectric constant of 2.6 at room temperature, to apply force on the dielectric sensor, as shown in Fig. 3(c). Two WR-90 rectangular waveguides operating in the frequency range from 8.2 GHz to 12.4 GHz were used as the excitation transmitter and receiver. The waveguides were connected to the input and output ports of a network analyser to measure the S_21_ parameter when different compressive forces were applied in the direction orthogonal to the sensor, as shown in Fig. 3(a).

**Figure 4:**
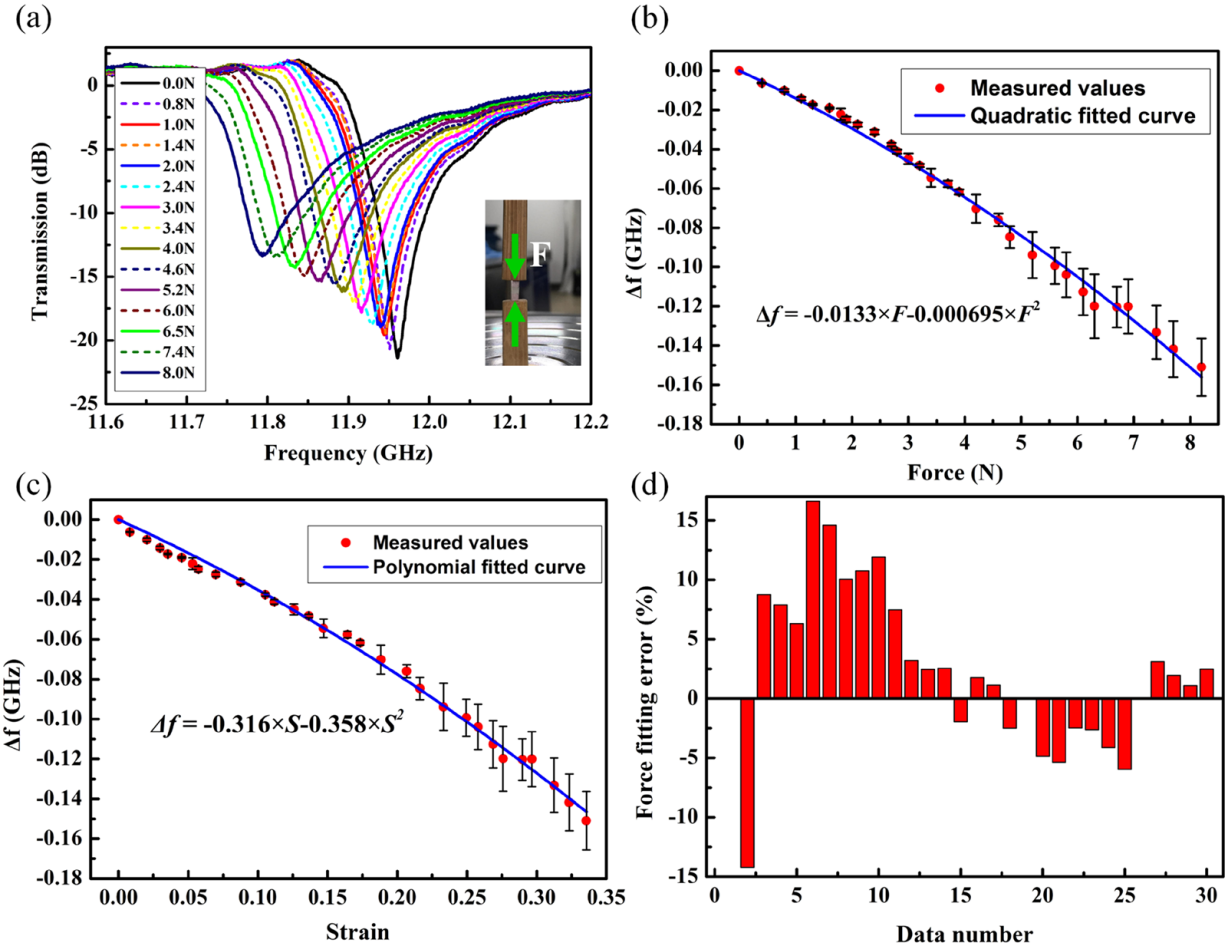
(**a**) The measured resonance frequency shift with different compressive force level applied on the sensor. (**b**) The measured relationship between the resonant frequency shift and the applied force. (**c**) The measured relationship between the resonant frequency shift and the strain of the sensor. (**d**) The errors that occurred between the measured values and the corresponding values that were calculated from the quadratic fitted Δ*f*–*F* curve.

The transmission spectra measured in S_21_ configuration of the dielectric sensor under different applied force magnitudes are shown in Fig. 4(a). A frequency shift was observed when the compressive force increased from 0.0 N to 8.0 N. For all of the measured loading levels, the transmission dips were greater than −12.5 dB, which made it straightforward to determine the frequency shift. Comparing the experimental and simulated results (shown in Figs 1(c) and 4(a), respectively), we can see that the measured transmission spectra of the sample agree well with the simulated spectra. Slight discrepancies result from the influence of the wood ejectors and fabrication errors. From the transmission curves shown in Fig. 4(a), we obtained the relationship between the resonance frequency shift and the applied compressive force. As in the simulation, we also fitted the measured values by using quadratic equations. The relationship between the frequency shift (Δ*f*) and the applied force (*F*) is shown in Fig. 4(b) while the relationship between Δ*f* and the strain of the sample (*S*) is shown in Fig. 4(c). We also calculated the errors that occurred between the measured values and the corresponding values that were calculated from the quadratic fitted Δ*f*–*F* curve. The sensor errors at the different data points were calculated as percentages and are shown in Fig. 4(d). From the figure, we see that the maximum fitting error for the data is less than 17%. It is well known that higher orders of fitted polynomials will produce more accurate fitting results. However, increasing the order of the polynomial will also produce a more complex calculation process. Therefore, we adopt the quadratic fitting procedure here to reach a compromise between the fitting accuracy and the calculation costs.

**Figure 5:**
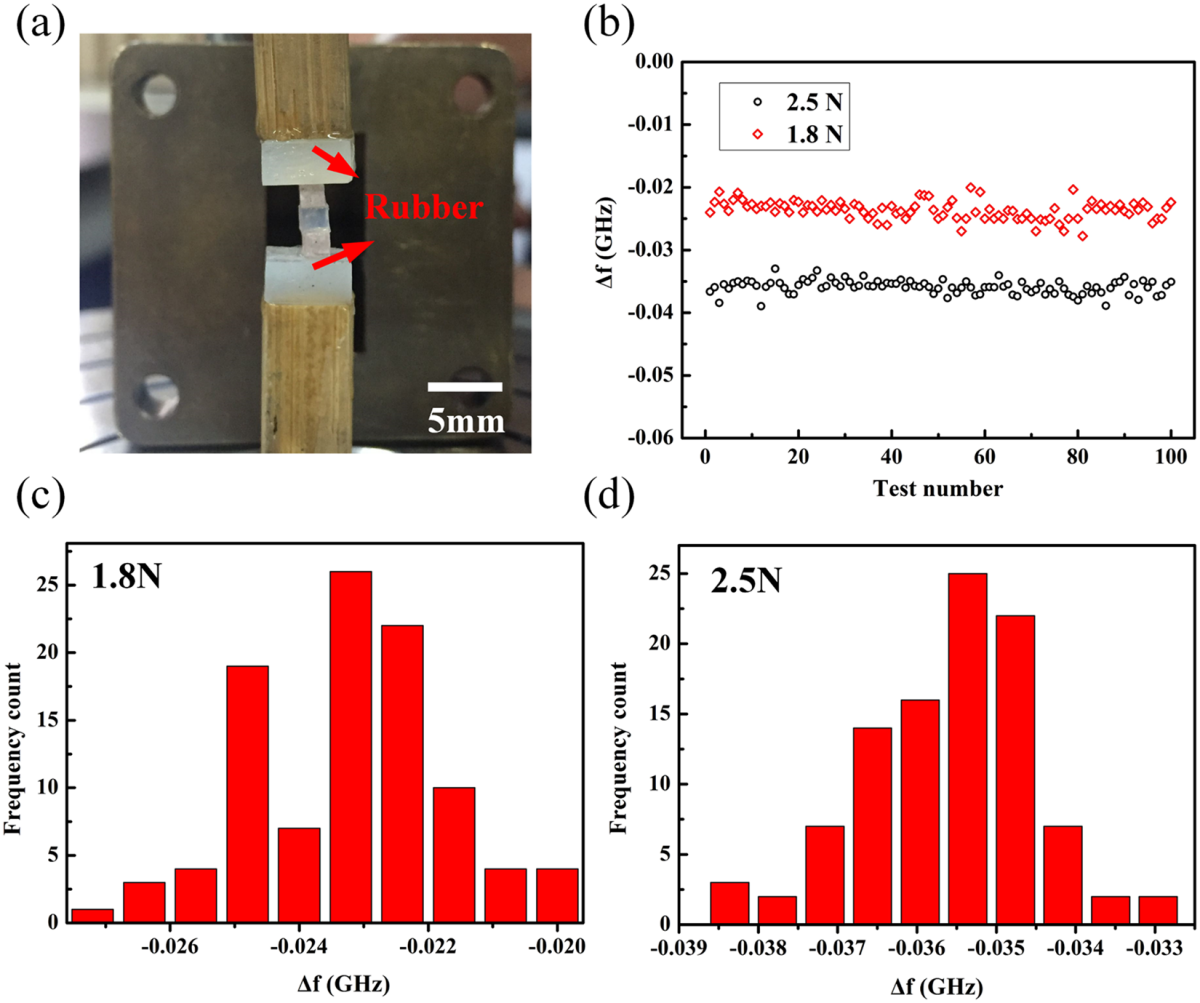
(**a**) Measurement setup for testing of the stability of the sample between soft materials. (**b**) Measured values of the frequency shift of the sensor for 100 repeated tests at loading levels of 1.8 N and 2.5 N. (**c**) Statistical distribution for the 100 repeated tests under a loading force of 1.8 N. (**d**) Statistical distribution for the 100 repeated tests under a loading force of 2.5 N.

To ensure that the sensor can be used within soft materials with high stability, we performed force tests by placing the sample inside soft materials. We stuck two pieces of rubber on to the wood ejector, and placed the sensor between these pieces, as shown in Fig. 5(a). Then, we performed loading and unloading processes 100 times at loading levels of both 1.8 N and 2.5 N, and recorded the resulting frequency shifts in the resonant frequency. The results are shown in Fig. 5(b), which illustrates the relatively good performance stability of the sensor. From the statistics shown in Fig. 5(c) and (d), we can conclude that under a compressive load of 2.5 N, the number of measured values of Δ*f* that are fluctuating around the average value within an error of 8% is 97, while the corresponding number for 1.8 N is 84; these results demonstrate the relatively good stability of the sensor. It should be noted here that after each loading and unloading process, we must wait for several seconds after each measurement performed using the sensor before the next test begins. Otherwise, measurement errors will be introduced by the viscoelastic properties of the rubber pieces.

## Discussion

The high sensitivity, biological compatibility and small size of the proposed dielectric force sensor make it suitable for many applications that require small forces and large strains, such as those that commonly occur inside soft materials like soft tissues and muscle, to be measured. The high sensitivity of the sensor described here is derived from the low elastic modulus of the silicone rubber, which undergoes greater mechanical deformation under the same applied load compared with materials such as silicon or Kapton tape, which have been used as substrates in previously studies^2, 16^. Of course, we do believe that with the continuing developments in materials engineering, new soft materials with greater flexibility, less viscoelasticity, and better biocompatibility are likely to be synthetized in the near future that would represent suitable replacements for the silicone rubber, and we will consider these materials.

Because the resonant frequency depends on the size and permittivity of the ceramic particles^9^, by carefully designing the size parameters of the sensor, or replacing the intermediate elastic layer with another elastomer with a different Young’s modulus, various dielectric sensors with tunable sensitivity and operating frequency region could be constructed to meet specific application demands. Compared with the previous sensors based on SRRs or metallic nanoparticle structures, this method provides a lower-cost and simpler approach to measuring force in soft biological materials.

## Conclusion

A dielectric force sensor with high sensitivity and small dimensions was designed by introducing an elastic material with low Young’s modulus into dielectric ceramic cubes. Experimental and simulation results show an approximate quadratic relationship between the frequency shift of the sensor and the applied compressive force with a maximum fitting error less than 17%. The sensor can be used over an ultra-large deformation strain range and has a relatively low operating frequency range in the microwave region. It should be noted that our current design cannot measure the shear strain. In our future work, we will extend our efforts to design new sensors that are capable of measuring the shear and normal strains simultaneously. Appropriate selection of the dimensions and permittivity of the dielectric resonators, as well as the Young’s modulus of the elastic layer, could enable the design of electromagnetic sensors with different sensitivity and measurement ranges for use in a variety of industrial applications.

## Methods

### Sample preparation

The ceramic dielectric was fabricated by solid-state reaction by mixing CaTiO_3_ powders with 2 wt%ZrO_2_. The dielectric cubes were achieved by cutting the dielectric ceramic plate into dimensions of 2 mm × 2 mm × 2 mm. The permittivity of the obtained ceramic was 115 + 0.001*i* at room temperature.

### Simulation and measurement

The samples were measured using two WR-90 rectangular waveguides with sectional sizes of 22.86 mm × 10.16 mm × 100 mm. The other ends of the two waveguides were connected to the input and output of a vector network analyzer (N5230C, Agilent Technologies, USA). The simulated microwave transmission S_21_ was achieved by using the Microwave Studio software package (CST Studio Suite 2016, Germany).

### Data Availability

The datasets generated during and analyzed during the current study are available from the corresponding author on reasonable request.

## Electronic supplementary material

Supporting information
