# Supplementary material for: A Mie resonant antenna with high sensitivity for force and strain measurement
Source: Sci Rep. 2017 Jul 4;7:4615. doi: 10.1038/s41598-017-04911-2 (PMC5496900; doi:10.1038/s41598-017-04911-2)
Supplement: Supplementary file 1 — Supporting information [file 41598_2017_4911_MOESM1_ESM.pdf]

Supporting information

A Mie resonant antenna with high sensitivity for force and strain measurement

Lingling Wu, [1] Xiaoqing Xi, [1] Bo Li, [2]* Ji Zhou[1]*

[1]State Key Laboratory of New Ceramics and Fine Processing, School of Materials Science and Engineering, Tsinghua University, Beijing 100084, China
[2]Advanced Materials Institute, Shenzhen Graduate School, Tsinghua University, Shenzhen, China

[1] *Author to whom correspondence should be addressed. Electronic mail: zhouji@mail.tsinghua.edu.cn

Author to whom correspondence should be addressed. Electronic mail: boli@mail.tsinghua.edu.cn

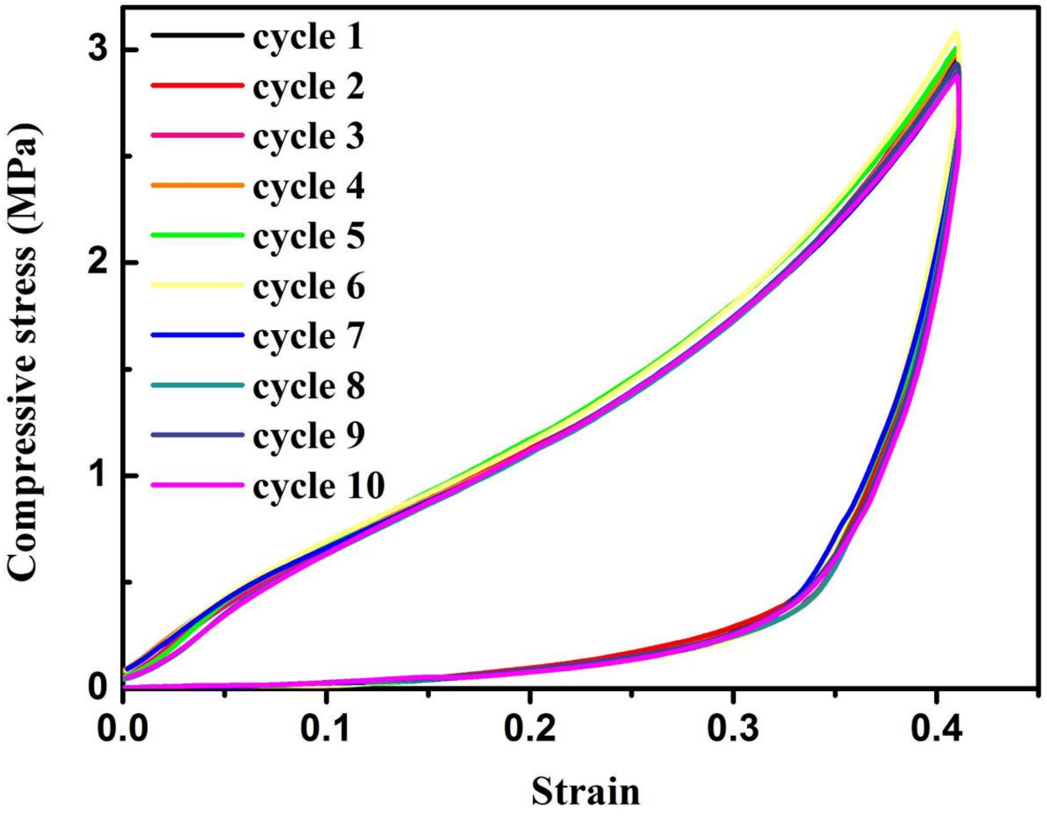

Figure S1 | 10 cycles of measured loading and unloading curves for the rubber
